# Adaptive Dynamic Subtidal Volume Ventilation (ADSV) Technology: Fluid Dynamic Principles and Physiologic Rationale for Personalized Treatment of Acute Respiratory Distress Syndrome

**DOI:** 10.3390/jcm15218714

**Published:** 2026-11-08

**Authors:** Aarthi Sekaran, Adel Bougatef, Gary Nieman

**Affiliations:** 1 SUNY Polytechnic Institute, Department of Civil and Mechanical Engineering, Utica, NY 13502, USA; 2 Independent Researcher, 1090 Brussels, Belgium; 3 SUNY Upstate Medical University, Department of Surgery, Syracuse, NY 13210, USA; niemang@upstate.edu

**Keywords:** acute respiratory distress syndrome, mechanical ventilation, adaptive dynamic subtidal ventilation, sliding venturi, respiratory system mechanics, pulmonary heterogeneity, alveolar recruitment

## Abstract

Acute respiratory distress syndrome (ARDS) imposes regionally heterogeneous, time-dependent alterations in pulmonary mechanics that fundamentally limit conventional ventilation strategies. Fixed-parameter approaches targeting global pressure, volume, or timing thresholds cannot simultaneously address the competing demands of alveolar recruitment and volutrauma prevention, nor can they adapt dynamically to the wide regional variation in compliance, resistance, and expiratory time constants that characterizes the injured lung. These limitations persist across volume-controlled, pressure-controlled, high-frequency oscillatory, and time-controlled ventilation strategies. This article provides a clinically oriented narrative review of the physiologic and fluid-dynamic principles underlying Adaptive Dynamic Subtidal Ventilation (ADSV), an emerging ventilation technology that incorporates a proximal airway device—the Sliding Venturi—that passively modulates subtidal gas delivery in response to evolving respiratory system mechanics. The device utilizes the Bernoulli and Venturi effects to establish a passive pressure–flow feedback mechanism in which downstream respiratory system mechanics influence gas entrainment, delivered subtidal volume, and airway pressure. In the low-compliance (high-elastance) ARDS lung, this may limit gas entrainment and subtidal volume delivery while maintaining distending pressure. As compliance improves, entrainment may increase, permitting larger subtidal volumes at lower pressures. This self-regulating behavior operates on millisecond timescales and adapts to changes in overall respiratory system mechanics. Patient-proximal placement facilitates rapid expiratory venting, potentially reducing circuit-related expiratory resistance and dynamic hyperinflation, although these effects require experimental validation. ADSV therefore provides a fluid-dynamic framework for mechanically responsive subtidal ventilation that may help manage the changing respiratory mechanics associated with ARDS. Bench and computational studies, followed by preclinical and clinical investigations, are needed to characterize device performance, evaluate regional physiological effects, and establish safety and efficacy in ARDS.

## 1. Introduction

**Figure 1:**
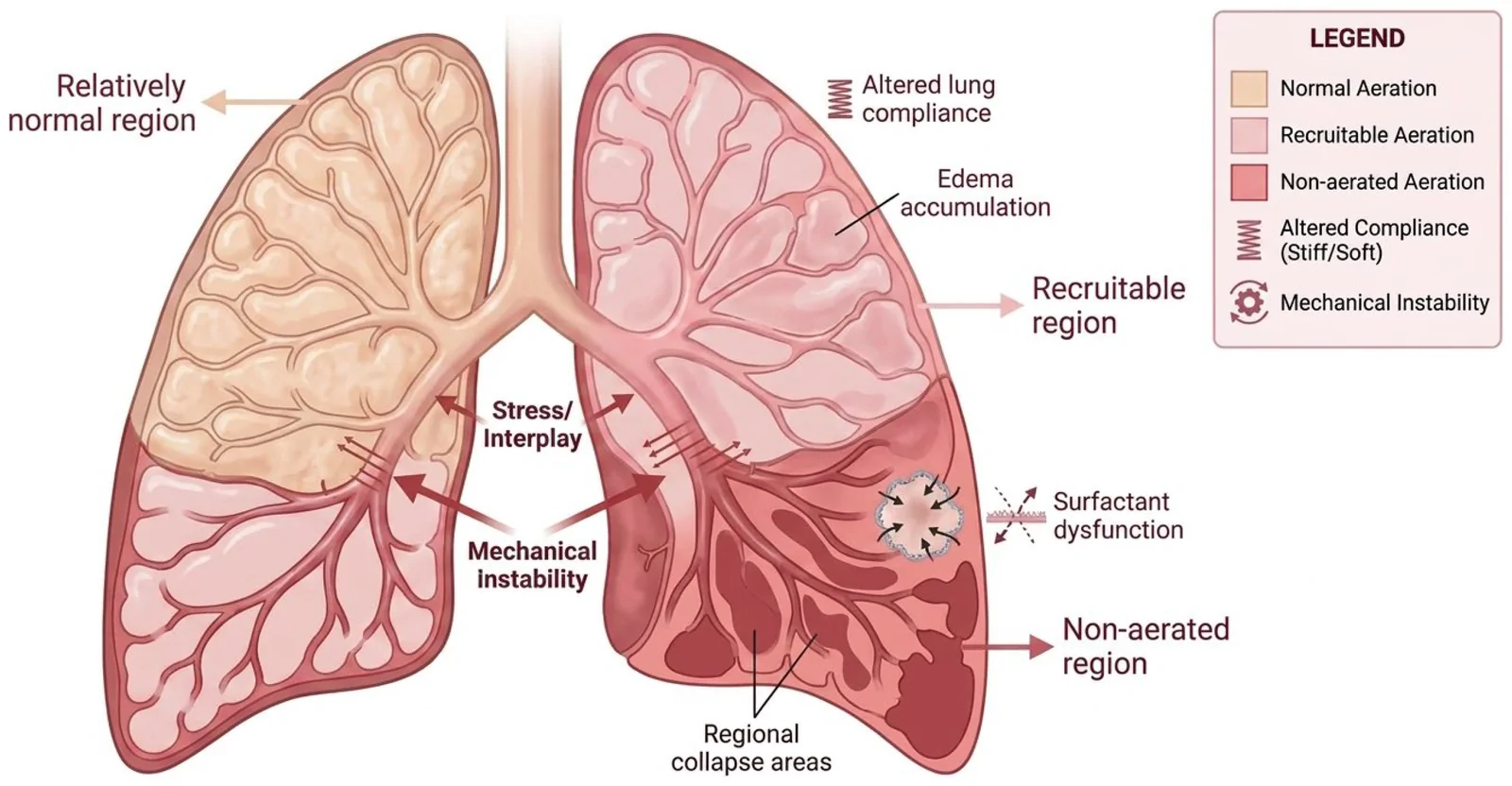
Spatial heterogeneity of ARDS pathology in lung cross-section.

Acute respiratory distress syndrome (ARDS) remains a significant challenge in critical care because of its complex pathophysiology and heterogeneous mechanical characteristics (Figure 1). The 2024 Global Definition of ARDS expands the Berlin criteria to include patients receiving high-flow nasal oxygen, pulse oximetry-based oxygenation assessment, and lung ultrasound as an alternative imaging modality. In the injured lung, regional collapse, edema, surfactant dysfunction, and reduced compliance contribute to spatially heterogeneous aeration and mechanical instability [1,2]. This creates a mechanically unstable system in which relatively normal, recruitable, and non-aerated lung regions may exist immediately adjacent to one another. As a result, the same applied airway pressure or tidal volume may have markedly different effects across the lung: some regions may remain collapsed, some may cyclically open and close, and others may undergo excessive distension. This regional heterogeneity is central to the pathophysiology of ventilator-induced lung injury (VILI). Mechanical stress can concentrate at interfaces between open and collapsed units, while repetitive recruitment and derecruitment can generate atelectrauma and local tissue injury [3,4,5].

**Figure 2:**
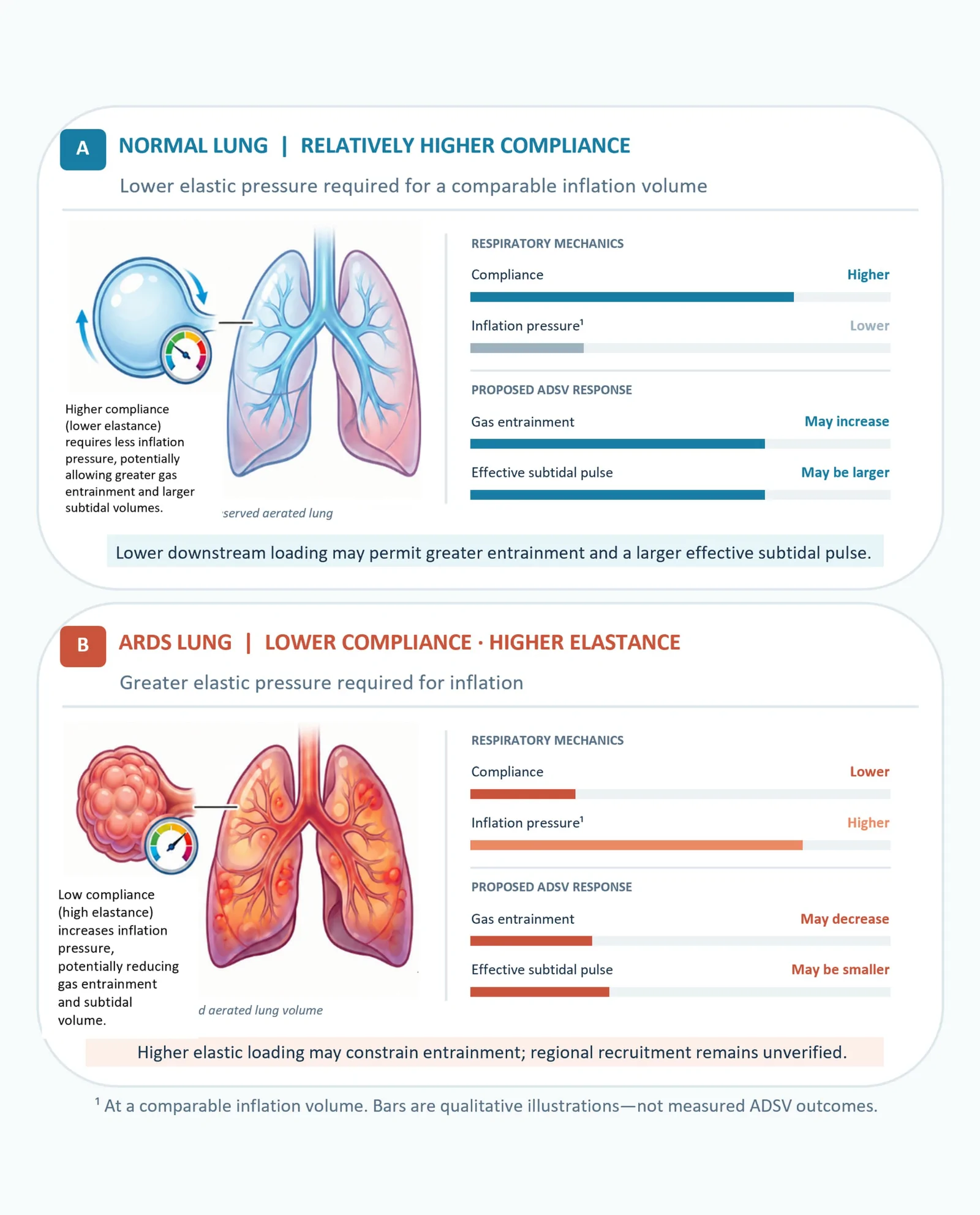
Conceptual comparison of respiratory mechanics in normal and ARDS lungs. Reduced compliance (increased elastance) in ARDS increases the pressure required for inflation, while airway resistance may provide an additional contribution to downstream pressure loading. The illustrated changes in gas entrainment and subtidal volume represent proposed ADSV behavior.

Lung-protective ventilation remains central to ARDS management, particularly through lower tidal volumes and limitation of plateau pressure. The landmark ARDS Network trial established the mortality benefit of lower tidal volume ventilation [6], while contemporary American Thoracic Society guidelines provide updated recommendations concerning positive end-expiratory pressure, neuromuscular blockade, corticosteroids, and extracorporeal support. Nevertheless, ventilation strategies must continue to balance recruitment, regional overdistension, and the injured lung’s changing mechanical properties (Figure 2) [7]. However, conventional modes remain limited by their reliance on global airway pressures, global tidal volumes, and fixed or semi-fixed timing parameters. These settings are typically adjusted based on whole-respiratory-system behavior, yet ARDS is fundamentally regional and dynamic. A tidal volume that is “protective” when indexed to predicted body weight may still be preferentially distributed to the remaining aerated “baby lung,” increasing local strain in vulnerable regions [3]. Similarly, pressure-controlled strategies may limit peak or plateau pressures globally while still failing to account for regional differences in opening pressure, collapse tendency, and time-dependent recruitment. As a result, ARDS-related mortality remains unacceptably high [8].

A major unresolved challenge is that the injured lung behaves as a viscoelastic system. Alveolar opening and closure depend not only on pressure magnitude; they also depend on how long pressure is applied and how long it is released. Injured alveoli may require sustained time at pressure to recruit, yet may collapse rapidly during expiration if expiratory duration is excessive relative to their regional emptying behavior. Thus, both inspiratory and expiratory timing are central to alveolar stability. The expiratory time constant, commonly defined as the product of respiratory system resistance and compliance, provides a clinically relevant framework for understanding how quickly lung units empty during expiration. In ARDS, regional differences in resistance and compliance produce a spectrum of local filling and emptying time constants [9,10]. Although these regional properties determine local ventilation behavior, their combined effects are reflected in the overall mechanical response measured at the airway opening.

This time-dependent behavior fundamentally limits conventional ventilation. If expiratory time is too long, unstable alveoli may derecruit before the next breath, promoting repetitive alveolar collapse and expansion (RACE). Increasing pressure or volume to counter derecruitment may overdistend already open lung units. As a result, clinicians must continuously balance recruitment against volutrauma, oxygenation against mechanical stress, and stabilization against excessive pressure or volume delivery. This balance is particularly difficult when ventilator settings are prescribed globally, but the disease process is regionally heterogeneous. Contemporary concepts of VILI increasingly recognize that injury risk is not determined by tidal volume or pressure alone, but by the combined mechanical load imposed on the lung, including pressure, volume, flow, respiratory rate, and timing [4,11]. Recent investigations have further suggested that respiratory system elastance may modify the mortality benefit associated with neuromuscular blockade in ARDS; however, the robustness and physiological basis of this treatment interaction remain debated, highlighting the limitations of relying on a single global mechanical parameter to predict therapeutic responsiveness [12].

These considerations underscore the need for ventilation strategies that respond to the injured lung’s dynamic and heterogeneous mechanical behavior. Conventional volume-controlled ventilation (VCV) ensures delivery of a prescribed tidal volume but may do so at the expense of variable airway and transpulmonary pressures as compliance and resistance change. Conversely, pressure-controlled ventilation (PCV) limits applied inspiratory pressure but allows tidal volume to vary with respiratory system mechanics. Although both approaches support lung-protective ventilation, neither independently controls regional gas distribution in response to spatial variations in compliance, resistance, or recruitment characteristics. This limitation is particularly relevant in ARDS, where the functional “baby lung” receives a disproportionate fraction of delivered ventilation and where stress and strain may be amplified at interfaces between aerated and non-aerated tissue [3,4]. Low-tidal-volume ventilation remains the evidence-based foundation of ARDS management, but it does not eliminate the competing risks of derecruitment, overdistension, and regional mechanical heterogeneity [6,13].

Several advanced modes have sought to address these limitations by incorporating different forms of physiologic adaptation. Adaptive Support Ventilation (ASV), for example, is a closed-loop mode that automatically adjusts tidal volume and respiratory rate based on measured respiratory mechanics and a target minute ventilation. In this respect, ASV illustrates an important conceptual shift away from fixed ventilator settings and toward breath-by-breath adjustment based on compliance, resistance, and expiratory time constant behavior [10]. However, ASV remains fundamentally organized around achieving a target minute ventilation through automated selection of tidal volume and respiratory rate; it does not directly address regional recruitment instability in a mechanically heterogeneous ARDS lung. Similarly, high-frequency oscillatory ventilation (HFOV) was developed to provide very small tidal volumes around a relatively constant mean airway pressure, theoretically limiting cyclic stretch while maintaining recruitment. However, large randomized trials in adults with ARDS did not show improved mortality with early HFOV compared with conventional lung-protective ventilation, underscoring that small tidal volumes alone are insufficient if mean pressure, hemodynamic burden, recruitment potential, and patient-specific mechanics are not appropriately balanced [14,15].

Other approaches, particularly the airway pressure release ventilation (APRV) mode set and adjusted using the time-controlled adaptive ventilation (TCAV) method, place greater emphasis on the time-dependent behavior of alveolar recruitment and derecruitment. These strategies recognize that the injured lung is not only pressure-dependent but also time-dependent: alveolar units may require sustained pressure to recruit and may collapse rapidly when expiratory time is excessive. TCAV, in particular, uses an extended inspiratory phase and a brief, mechanically guided expiratory release to promote recruitment while limiting end-expiratory derecruitment. Expiratory duration is personalized based on lung mechanics, measured as the slope of the expiratory flow curve. This framework is highly relevant to ARDS because regional differences in resistance and compliance generate different filling and emptying time constants, meaning that lung units do not inflate or deflate synchronously under conventional global ventilator settings [9,16,17].

ADSV is an emerging personalized ventilation technology that fits within the broader movement toward mechanically responsive ventilation, while remaining distinct from VCV-, PCV-, ASV-, HFOV-, and APRV-based strategies. Rather than relying primarily on fixed tidal volume delivery, fixed inspiratory pressure, a target minute ventilation algorithm, or oscillation around a sustained mean airway pressure, ADSV delivers rapid, subtidal gas pulses with pressure–flow behavior that responds dynamically to the evolving resistance and elastance of the respiratory system. Conceptually, ADSV seeks to modulate gas delivery in response to changing respiratory system mechanics. Increased downstream pressure loading, particularly from elevated elastance in ARDS, is expected to reduce entrainment and constrain subtidal volume delivery, while improved compliance may permit greater entrainment. In this framework, ADSV may offer a mechanically responsive approach to ventilating compromised lungs by incorporating the viscoelastic and time-dependent nature of alveolar recruitment and derecruitment into the breath-delivery pattern itself. This distinction is important because ARDS ventilation is increasingly understood not only as a problem of selecting safe global pressures and volumes, but also as a problem of matching pressure, flow, volume, and timing to a mechanically heterogeneous and continuously changing lung.

The fundamental ADSV concept, including the Sliding Venturi mechanism and its proposed physiological applications, was previously described by [18]. Building on that foundation, this article offers a focused fluid-dynamic and clinical interpretation of ADSV, emphasizing downstream pressure–flow coupling, respiratory mechanical responsiveness, and the experimental requirements needed to validate the proposed physiological effects. The goal is not to establish clinical efficacy but to critically examine the proposed fluid-dynamic mechanisms, distinguish established physical principles from unvalidated physiological hypotheses, and identify measurable outcomes needed for future experimental and clinical evaluation.

## 2. Conceptual Architecture and Operational Rationale of ADSV

### 2.1. Relationship to High-Frequency Percussive Ventilation

ADSV fits conceptually within the broader family of high-frequency, subtidal-volume ventilatory strategies, particularly high-frequency percussive ventilation (HFPV). HFPV combines high-frequency mini-bursts with convective ventilation, superimposing rapid pulsatile gas delivery on a lower-frequency pressure–flow pattern. Researchers have investigated this approach in acute respiratory failure and ARDS because it may improve gas exchange while limiting large cyclic tidal excursions, though clinical evidence remains limited and heterogeneous [19,20,21]. Godet et al. [20] specifically characterize HFPV as combining diffusive high-frequency mini-bursts with convective ventilation patterns, and report increased alveolar recruitment in early ARDS on physiologic CT-based assessment.

ADSV shares the general HFPV principle of repeated small-volume gas pulses rather than conventional tidal-volume cycling, but should not be presented as simply another HFPV variant. ADSV’s distinguishing feature is coupling rapid subtidal pulses to a mechanically responsive delivery system in which downstream respiratory-system resistance and elastance directly influence delivered pulse volume and pressure—positioning ADSV as an adaptive pressure–flow delivery concept rather than a purely frequency-based mode. This distinction matters because prior experience with high-frequency strategies, including HFOV, demonstrates that small tidal volumes alone do not ensure lung protection; clinical benefit depends on how pressure, volume, timing, recruitment, and hemodynamic effects are integrated in the injured lung [14,15,22].

### 2.2. Core Components and Adaptive Pressure–Flow Behavior

The core architecture of the ADSV method of interest in this paper consists of a gas-pulse generator coupled to a sliding-venturi assembly with an entrainment chamber. The system delivers rapid gas pulses on the millisecond timescale, generating repeated subtidal-volume injections into the airway. Critically, the entrainment port permits additional gas to be drawn into the delivered pulse stream, so the effective subtidal volume is determined not solely by the primary driving pulse but by the mechanical conditions downstream in the respiratory system. In a relatively compliant, low-resistance lung, downstream impedance is low, permitting greater entrainment augmentation and larger effective subtidal delivery. In a low-compliance lung, higher inflation pressure may reduce the pressure gradient driving entrainment, limiting subtidal volume delivery while allowing distending pressure to develop.

This architecture is intended to address changes in respiratory system mechanics during ventilation of the heterogeneous ARDS lung. Although regional pulmonary units exhibit different compliance, resistance, and recruitment characteristics, the proposed ADSV mechanism responds to their combined downstream mechanical load. Consequently, the resulting breath-delivery pattern may vary as respiratory system impedance changes without independently controlling individual lung regions.

### 2.3. Breath-Delivery Philosophy

ADSV’s breath-delivery philosophy is grounded in the recognition that ARDS is not only a pressure- and volume-dependent disease, but also a fundamentally time-dependent mechanical process. Regional alveolar units may require sustained distending conditions to recruit, yet derecruit rapidly when expiratory duration exceeds their local time constants. Contemporary time-controlled ventilation frameworks confirm that alveolar stability depends on the interaction among pressure, volume, resistance, compliance, and expiratory duration, rather than on any single ventilator parameter in isolation [16,17].

Millisecond-scale subtidal pulses are intended to promote gas transport while limiting large cyclic tidal excursions. Mechanisms such as convective transport, pendelluft, longitudinal dispersion, and asymmetric velocity profiles may contribute to gas exchange, as described for other high-frequency ventilation strategies [22]. In ADSV, the proposed additional feature is modulating gas entrainment through downstream respiratory mechanics; its influence on regional ventilation and gas exchange remains to be demonstrated. This provides a mechanistic rationale grounded in device architecture and applied physiology. Bench, imaging, computational, animal, and clinical studies are needed to quantify ADSV pressure–flow behavior, regional ventilation distribution, recruitment effects, and safety under ARDS-relevant conditions before establishing therapeutic efficacy.

## 3. ADSV Device Anatomy and Function

The ADSV device is an integral part of the setup and primarily delivers these short subtidal pulses. The device is a comprehensive unit that integrates a ventilator, humidification system, and breathing circuit (composed of a sliding venturi and multi-lumen tubing for inspiration and expiration). The proposed setup would place this device immediately proximal to the endotracheal tube (ETT). Of the included components, the ventilator unit provides the primary gas source, flow-control mechanism, humidification, and pressure–flow–oxygen monitoring.

The sliding venturi assembly is positioned close to the patient interface, directly upstream of the ETT and lungs. This near-patient placement matters because it lets the ADSV modulate gas delivery closest to the respiratory system, minimizing circuit-related losses and enabling the delivered subtidal pulses to interact directly with the mechanical impedance of the patient’s airways and injured lung. Thus, while the gas supply line maintains conventional support functions such as gas conditioning, monitoring, and feedback control, the ADSV device serves as the patient-proximal flow-modulating element that shapes the final breath-delivery pattern entering the ETT.

The ADSV Patient Interface Device integrates a gas interrupter valve and a Sliding Venturi assembly positioned near the patient’s airway. The driver supplies pressurized gas, which is converted into rapid pulses by the interrupter valve. The Sliding Venturi incorporates an axially movable element that changes the internal flow configuration during inspiration and expiration. Previously described embodiments employed pneumatic or electromagnetic actuation to control this movement, allowing the coordinated delivery of inspiratory gas pulses and expiratory venting.

The primary gas supply may consist of air, oxygen, or a prescribed mixture delivered through a blending system. The control unit regulates the primary gas pulses and associated flow pathways, while secondary gas entrainment contributes to the effective subtidal volume. The delivered oxygen concentration therefore depends on the composition and relative contributions of the primary and entrained gas streams. Downstream of this circuit, the sliding venturi assembly is positioned close to the patient interface, directly upstream of the ETT and lungs. This near-patient placement is important since it allows the ADSV device to manage the combined subtidal breath, consisting of both bias and pulse flow, at the point closest to the respiratory system. In this configuration, the ventilator assembly module handles gas conditioning, monitoring, feedback control, and pneumatic actuation, while the sliding venturi module serves as the patient-proximal flow-modulating element. The patient-proximal arrangement permits the delivered pulses to interact directly with downstream respiratory impedance. Because the assembly incorporates moving components and communicates with the patient airway, condensation and retained secretions may influence valve mobility and flow characteristics. Patent-described configurations incorporate disposable components and, in some embodiments, monitoring for impaired shuttle movement. However, resistance to secretion accumulation and moisture-related malfunction requires device-specific evaluation. The proposed ADSV system incorporates three operating modes: Therapy, Bilevel, and Hybrid. Therapy Mode delivers rapid subtidal pulses around a single pressure level and is intended for ventilatory support and airway clearance. Bilevel Mode provides two adjustable pressure levels with clinician-selected durations, along with control of pulse size, frequency, and timing. Hybrid Mode combines Bilevel ventilation with programmed intervals of Therapy Mode. The operator interface is designed to display ventilation parameters and associated pressure, flow, volume, timing, and oxygen alarms. These operating modes represent device capabilities rather than established clinical protocols for ARDS. The described control architecture permits adjustment of primary gas delivery, pulse frequency and duration, and the timing of the larger inspiratory and expiratory phases. The effective subtidal volume depends on the interaction between the primary gas stream, entrainment, and downstream respiratory mechanics rather than a fixed tidal volume.

**Figure 3:**
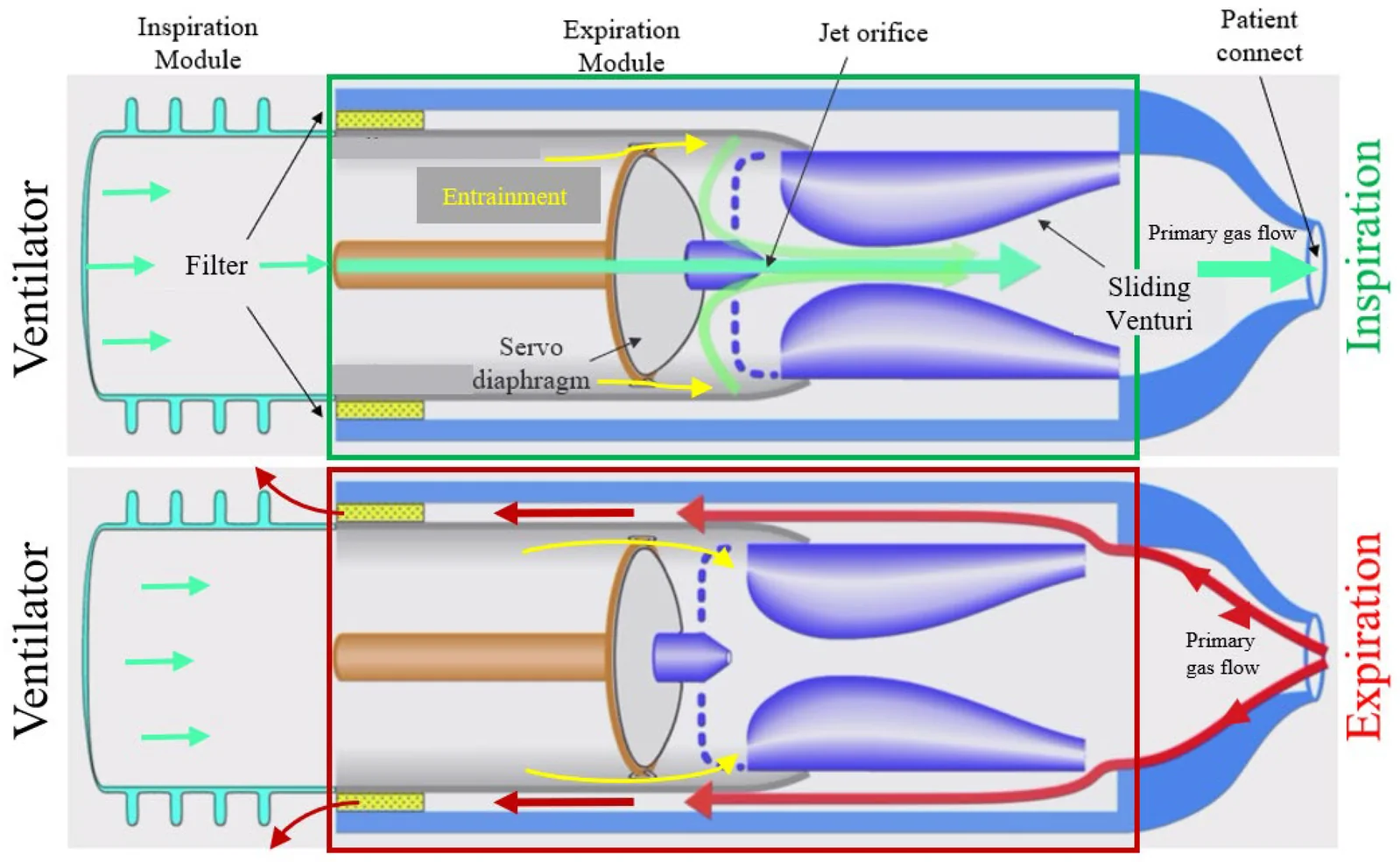
Conceptual cross-sectional schematic of the ADSV patient-interface assembly during inspiration (**upper panel**) and expiration (**lower panel**). During inspiration, the primary gas jet generates a low-pressure region that promotes secondary gas entrainment (yellow arrows), augmenting the subtidal gas pulse delivered toward the patient. During expiration, the movable assembly provides a pathway for returning gas to exit through the expiratory ports (red arrows). The degree of entrainment is influenced by downstream respiratory system pressure loading. Device movement and flow pathways are shown schematically.

**Figure 4:**
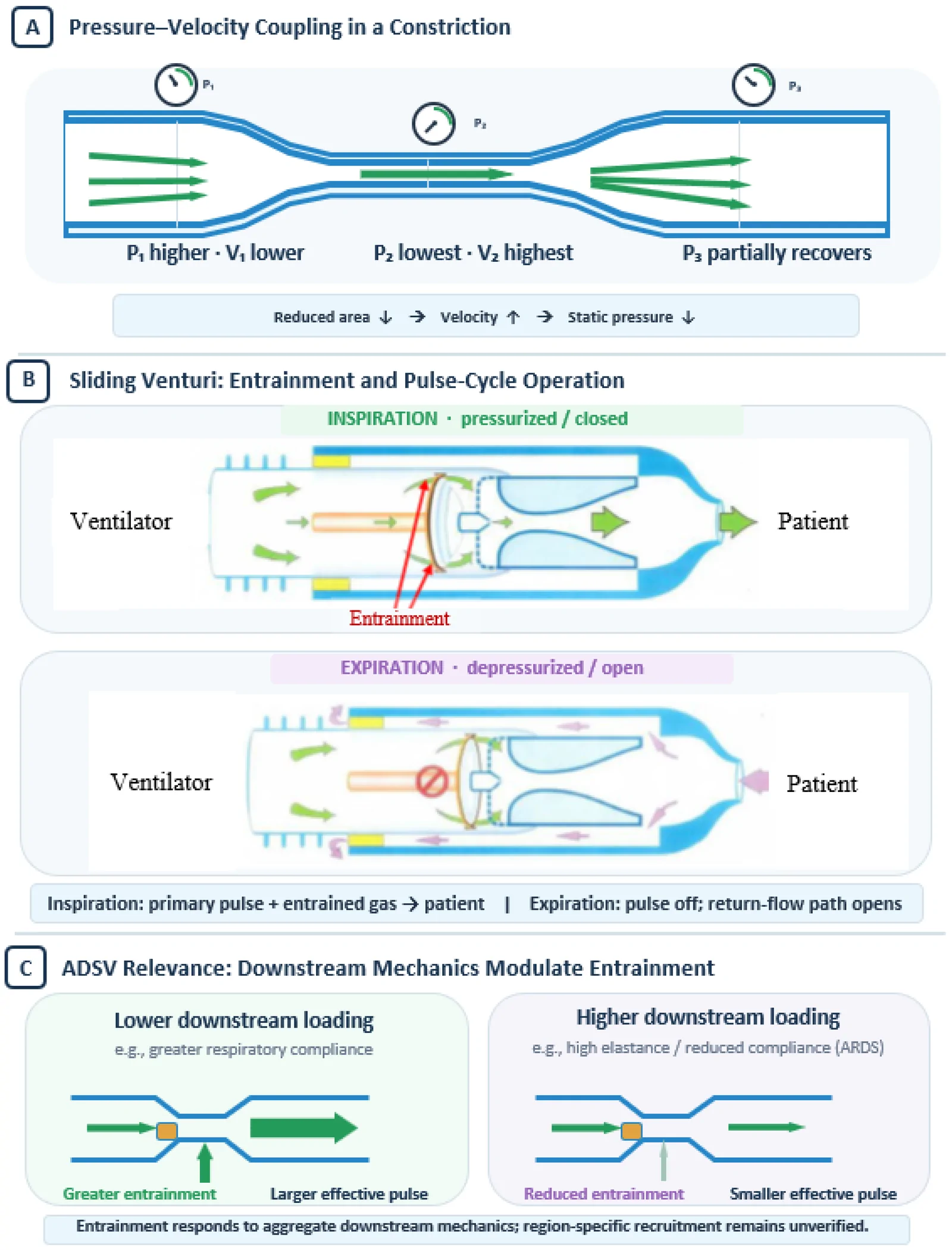
Conceptual illustration of pressure–velocity coupling, jet entrainment, and downstream impedance effects relevant to the ADSV sliding venturi mechanism. (**A**) In a simplified constricted flow passage, reducing the cross-sectional area increases gas velocity at the throat and locally reduces static pressure, followed by partial pressure recovery downstream. (**B**) Conceptual representation of secondary gas entrainment into a primary high-velocity jet. The entrainment port corresponds to the secondary gas-entry openings surrounding the primary jet in the patient-interface assembly shown in Figure 3. (**C**) Proposed modulation of gas entrainment by downstream respiratory pressure loading. Lower downstream loading may promote greater entrainment, whereas elevated elastic or resistive loading may reduce entrainment and constrain effective subtidal pulse volume. The entrained gas composition depends on the device’s secondary gas source and may influence the delivered inspired oxygen fraction. This passive coupling between entrainment behavior and respiratory system impedance provides a conceptual basis for mechanically responsive subtidal gas delivery.

Figure 3 illustrates the proposed patient-interface assembly during inspiration and expiration. During inspiration, the primary gas pulse passes through the jet orifice into the Venturi mixing region. The resulting low-pressure region promotes secondary gas entrainment through the inlet openings surrounding the jet, corresponding to the entrainment port illustrated conceptually in Figure 4B. The primary and entrained gas streams mix before passing through the downstream Venturi section toward the patient connection. During expiration, the primary gas pulse ceases or decreases, and the movable assembly allows gas to return from the patient through the expiratory pathway. The patient-proximal outlet is designed to facilitate rapid pressure release. However, expiratory pressure and the degree of lung emptying depend on expiratory timing, outlet resistance, and respiratory system mechanics; therefore, the absence of intrinsic PEEP or dynamic hyperinflation cannot be assumed. A key feature of this flow regime is the potential for simultaneous inspiratory and expiratory gas transport within the airway, rather than the temporally separated, predominantly unidirectional flow characteristic of conventional mechanical ventilation. Supplementary Video S1 illustrates the hypothesized countercurrent flow pattern and its conceptual distinction from the sequential inspiratory and expiratory flow characteristic of conventional mechanical ventilation.

The proposed adaptive response arises from the interaction between jet-induced entrainment and downstream respiratory pressure loading. In a low-compliance lung, elevated inflation pressures may reduce the entrainment-driving pressure gradient, constraining the effective subtidal volume. As respiratory system compliance improves, entrainment may increase. This pressure–flow coupling provides a passive mechanism for modulating delivered gas volume without requiring regional lung measurements.

## 4. Fluid Dynamic Principles Underlying ADSV: The Bernoulli and Venturi Effects

The proposed adaptive behavior of ADSV is grounded in the Bernoulli and Venturi effects and their interaction with downstream respiratory pressure loading. These principles explain how downstream respiratory mechanics can passively influence gas entrainment within the Sliding Venturi, independently of the electronic controls responsible for primary gas delivery and pulse timing. In the following discussion, upstream refers to the ventilator-side primary gas supply, whereas downstream refers to the patient-directed flow pathway during inspiration. Expiratory gas follows the reverse direction toward the device’s exhalation ports.

The Bernoulli effect describes how pressure changes when a gas accelerates through a constriction. As gas enters a narrowed section of tubing, its forward velocity must increase to maintain flow continuity. Under idealized steady-flow conditions, increased velocity through a constriction is accompanied by a decrease in static pressure, consistent with Bernoulli’s principle (Figure 4A). The faster the gas moves, the lower the pressure it exerts against the tube walls. In practical terms, progressively narrowing the tube diameter from a wide upstream segment produces a stepwise fall in wall pressure proportional to the degree of constriction. This phenomenon has direct implications for airway mechanics, as analogous pressure-velocity relationships govern gas behavior in branching pulmonary airways during both normal and disease states [23,24].

The Venturi effect extends this principle by introducing a secondary port at the point of maximum constriction—the throat (Figure 4B). Because the wall pressure at the throat falls below atmospheric pressure, gas from the surrounding environment is passively drawn inward through this port, augmenting the primary flow stream. The total gas volume exiting the downstream end of the tube is therefore greater than what entered at the upstream end. The magnitude of this entrainment depends on the pressure differential between the throat and the environment: the lower the throat pressure relative to ambient, the greater the entrained volume [25,26,27,28]. Venturi-based gas entrainment is established in respiratory devices, including oxygen delivery and aerosol generation systems [24]. Its application to mechanically responsive subtidal gas delivery has also been described in earlier ADSV-related device disclosures. The present analysis focuses on the pressure–flow interactions underlying this proposed application and their implications for respiratory mechanical loading [18].

Because the effective subtidal volume includes both primary and entrained gas, the delivered inspired oxygen fraction depends on the oxygen concentrations and relative flow contributions of these streams. If ambient air is entrained, variations in entrainment may change the delivered FiO_2_, even with a constant primary gas composition. Accurate oxygen delivery therefore requires characterizing the gas-mixing process and appropriately monitoring or controlling the final inspired oxygen concentration. The ADSV system incorporates an electronically controlled oxygen blender designed to regulate the oxygen concentration of both primary pulse gas and bias flow over a range of 21–100% FiO_2_. The breathing-circuit configuration is designed to supply the entrained gas at the same oxygen concentration as the primary gas stream. Under this condition, variations in the relative entrainment fraction would not independently alter the delivered FiO_2_. Nevertheless, patient-proximal oxygen monitoring and experimental validation are necessary to confirm the accuracy and stability of oxygen delivery across changing respiratory mechanics.

For a simplified, fully mixed flow, this dependence can be written as

$$F_{IO_{2},\;delivered}=\frac{\dot{V_{p}}F_{IO_{2},\;p}+\dot{V_{e}}F_{IO_{2},\;e}}{\dot{V_{p}}\dot{{+V}_{e}}}$$

where the subscripts p and e represent primary and entrained gas streams, respectively.

In the Sliding Venturi module, two coupled pressure–flow responses occur simultaneously and act in opposition. The first is the entrainment response described above: as the primary gas pulse accelerates through the venturi throat, local pressure within the entrainment region falls, drawing additional gas into the flow stream and augmenting the subtidal pulse delivered toward the patient. The second is a downstream impedance response. As the delivered flow enters the patient’s airways, part of the flow energy is converted into the pressure required to inflate and recruit the lung. The pressure required for respiratory inflation includes both elastic and resistive contributions. Increased respiratory system elastance, as commonly observed in ARDS, increases the pressure required for a given lung volume, while airway resistance contributes additional pressure loss during gas flow. The resulting downstream pressure loading may increase pressure within the entrainment region, reducing the available entrainment-driving pressure gradient and constraining the effective subtidal volume. The effective entrainment, and therefore the effective subtidal volume delivered to the patient, is determined by the balance between these opposing pressure–flow effects during each pulse cycle. When the entrainment-driving pressure differential exceeds the retrograde pressure transmitted from the airways, additional gas is entrained, and pulse volume increases. Conversely, when downstream airway pressure rises because of increased resistance or elastance, the entrainment gradient is reduced, limiting pulse augmentation and constraining delivered subtidal volume.

The proposed pressure–flow response provides a basis for several experimentally testable predictions concerning device operation and respiratory physiology. Table 1 summarizes the principal mechanisms, measurable quantities, and validation methods required to establish their relevance to ADSV.

The clinical consequence is a mechanical self-regulation that is inherently matched to lung pathophysiology (Figure 4C). In a lung with low resistance and normal compliance, backpressure into the venturi chamber is low, the entrainment gradient predominates, and a relatively large subtidal volume is delivered at low airway pressure. The proposed clinical relevance of this mechanism lies in its responsiveness to changing respiratory system mechanics (Figure 4C). In a relatively compliant lung, lower downstream pressure loading may permit greater entrainment and larger subtidal volumes. Conversely, the elevated elastance characteristic of ARDS may increase downstream pressure loading, suppress entrainment, and constrain volume delivery. Although this passive pressure–flow response is expected to occur over individual pulse cycles, its capacity to improve alveolar recruitment or limit regional overdistension remains to be demonstrated experimentally.

This behavior mirrors the physiologic principle that lung protection in ARDS requires individualized pressure-volume matching rather than uniform targets across mechanically heterogeneous tissue [29,30,31]. The critical distinction from conventional ventilation is that in ADSV, this matching occurs passively, at millisecond timescales, driven entirely by the fluid mechanics of the Venturi geometry rather than by clinician titration or closed-loop algorithms.

## 5. Adaptive Volume and Pressure Delivery Across Lung Pathophysiology

The clinical translation of the Bernoulli and Venturi principles described above is best understood by tracing gas delivery behavior across two contrasting lung states: the mechanically normal lung and the injured ARDS lung.

In a relatively compliant lung, lower downstream pressure loading is expected to permit greater gas entrainment and larger effective subtidal volumes. As lung inflation progresses, increasing elastic recoil may raise downstream pressure loading and progressively reduce entrainment. The magnitude of this response depends on the device operating conditions and respiratory system mechanics. Gas inflow and outflow equilibrate gradually during the inspiratory accumulation phase, consistent with the progressive filling behavior of a compliant lung across a wide range of regional time constants [32].

In ARDS lungs, this trajectory is fundamentally altered by the mechanical properties of the injured parenchyma. The ARDS lung is not simply a smaller or stiffer version of the normal lung; it is a structurally heterogeneous organ in which regions of near-normal compliance coexist with collapsed, consolidated, and fibrotic tissue—the so-called “baby lung” concept [33]. In ARDS, the reduced volume of aerated lung tissue and elevated respiratory system elastance increase the pressure required for inflation. As lung volume increases, this elastic pressure loading may reduce the pressure differential driving entrainment, thereby constraining effective subtidal volume delivery. Although such behavior could potentially limit excessive volume delivery, its influence on regional inflation and overdistension requires direct experimental evaluation [34,35].

The proposed entrainment mechanism may constrain delivered subtidal volume while still allowing distending airway pressure to develop. Whether this pressure is sufficient to recruit collapsed lung regions without causing overdistension elsewhere depends on regional opening pressures, compliance, and applied pressure duration. As overall respiratory compliance improves, reduced downstream pressure loading may permit increased entrainment and larger subtidal volumes.

ADSV is proposed to generate progressive pressure and volume accumulation through the delivery of successive subtidal pulses, with incomplete expiration between pulses, rather than a single large tidal inflation [31,36]. This behavior may produce different inflation responses in lung regions with varying compliance, opening pressures, and recruitment characteristics. However, the extent to which this pressure accumulation promotes recruitment of atelectatic regions without overdistending normally aerated tissue remains unverified [16,17,18]. Direct measurements of regional inflation and tissue deformation are required to evaluate this proposed protective mechanism. Patient-proximal placement may reduce circuit-related gas compression effects and facilitate rapid expiratory venting. Nevertheless, expiratory flow limitation, residual alveolar pressure, and dynamic hyperinflation depend on expiratory timing and respiratory system mechanics and require experimental characterization.

## 6. Gas Flow Dynamics in the Pulmonary Microenvironment

Beyond the macroscopic pressure-volume behavior described above, ADSV technology is proposed to generate a distinctive intrapulmonary gas flow pattern that may have physiologic relevance beyond simple tidal gas exchange. This section outlines the conceptual framework for this behavior and explicitly presents it as a mechanistic hypothesis requiring experimental validation.

**Figure 5:**
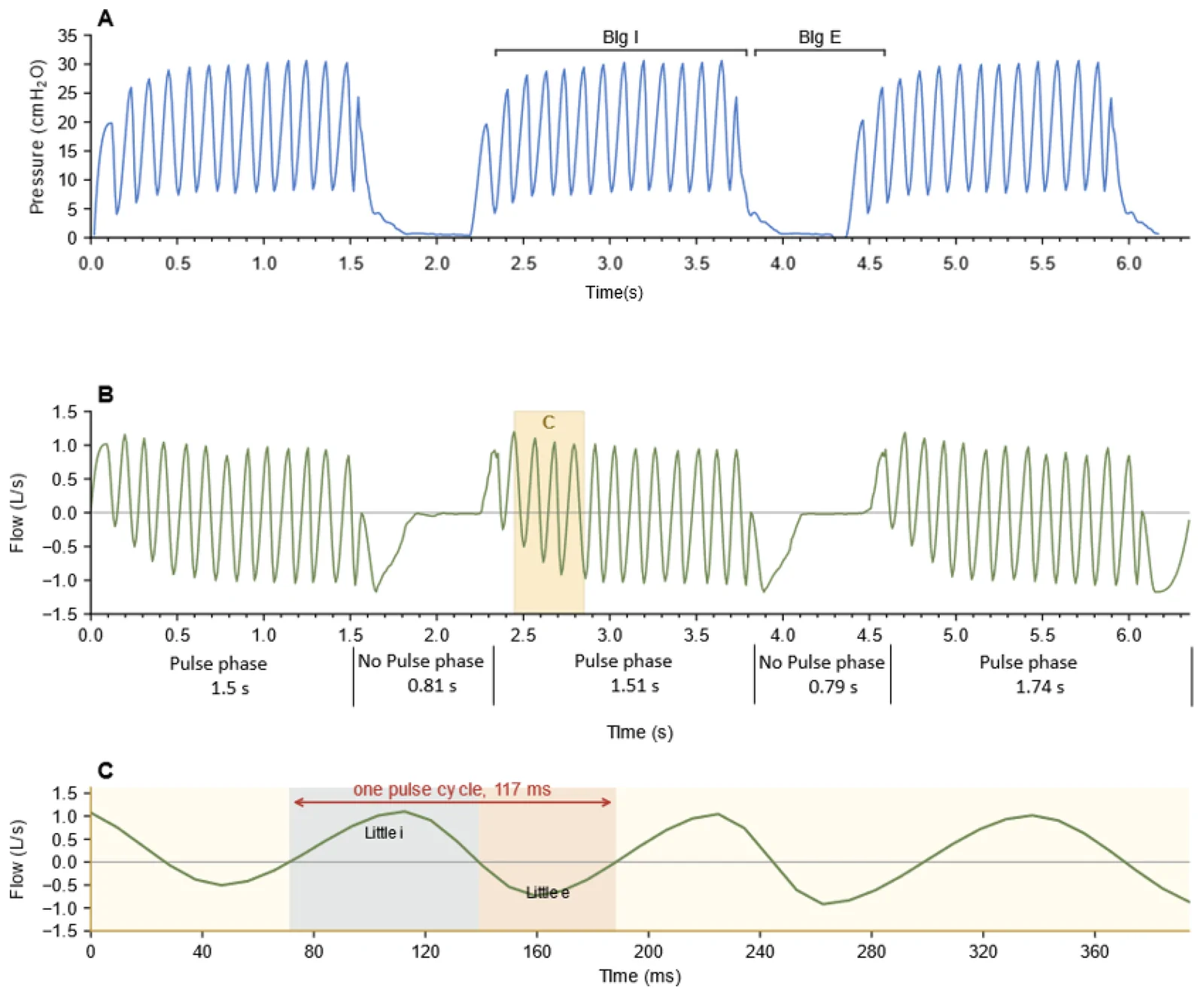
Airway pressure and flow during ADSV delivery, showing the high-frequency pulse train that generates the convective waveform. (**A**) Airway pressure. (**B**) Airway flow; positive is inspiratory. The macro-breath alternates between a pulse phase (Big I), during which successive sub-tidal pulses progressively raise airway pressure and lung volume to an oscillatory plateau, and a no-pulse phase (Big E), during which pulses stop and the lung empties passively. (**C**) Detail of the boxed region in (**B**), showing individual pulses. Each pulse comprises an inspiratory phase (Little i) and an expiratory phase (Little e). The illustrated pulse rate is 500 cycles/min (approximately 120 ms per cycle), with a pulse inspiratory duration of 30–50 ms and expiratory duration of 50–80 ms. The waveform illustrates a configuration with no externally prescribed PEEP at the exhalation port; alveolar end-expiratory pressure cannot be inferred from this boundary condition alone. Waveform representation adapted from [18].

ADSV delivers gas as a high-frequency percussive breath, typically set to generate a sinusoidal convective waveform at frequencies between 30 and 1200 cycles per minute, superimposed on a lower-frequency respiratory cycle of 0 to 70 breaths per minute. Each macro-breath consists of a sequence of high-frequency, subtidal percussive pulses superimposed on a slower respiratory cycle. At the pulse level, each percussion comprises a brief inspiratory phase (“little i”) followed by an expiratory phase (“little e”) (Figure 5). During the macro-inspiratory phase (“Big I”), successive subtidal pulses are delivered before the preceding pulse has completed. This produces a progressive, stepwise accumulation of gas volume within the respiratory system, with corresponding increases in airway pressure and lung volume. As the Big I progresses, the incremental volume contributed by successive pulses is balanced by the volume leaving during the intervening little-e phases, producing a dynamic oscillatory plateau rather than a static end-inspiratory state. Thus, pressure and volume continue to fluctuate at the percussive frequency even after the slower macro-inspiratory envelope has approached its plateau.

At the transition to the macro-expiratory phase (“Big E”), the sustained inspiratory drive is released and stored elastic energy within the respiratory system promotes passive lung emptying. This longer expiratory interval provides the main pathway for removing accumulated alveolar gas and CO_2_, while the superimposed high-frequency pulsations during the respiratory cycle provide additional opportunities for intrapulmonary gas transport and mixing. Pulse-scale ventilation refers to the rapid delivery and partial expiration of individual subtidal gas pulses within the larger respiratory cycle. Although individual pulse volumes may be smaller than anatomical dead space, cumulative gas transport and mixing across successive pulses may contribute to effective ventilation. Similar to other high-frequency ventilation strategies, effective gas transport may arise from the combined effects of bulk convective transport over the macro-breath, asymmetric inspiratory and expiratory velocity profiles, enhanced longitudinal dispersion (including Taylor-type dispersion), interregional gas redistribution or pendelluft, and cardiogenic mixing. Consequently, ADSV can be viewed as operating simultaneously on two characteristic timescales: a high-frequency percussive timescale that promotes intrapulmonary transport and mixing, and a lower-frequency macro-breath timescale that governs progressive lung inflation and subsequent expiratory emptying [22,37,38,39].

The distinctive hypothesis associated with ADSV is that its high-energy pulsatile flow may generate simultaneous bidirectional gas movement within individual airways—a continuous “river” of gas in which fresh gas travels centrally into the airway lumen while outgoing gas returns along the airway walls, as hypothesized by [18]. A conceptual analogy is water forced into a glass: as jet velocity exceeds a threshold, inflow and outflow occur simultaneously rather than sequentially. If this bidirectional pattern operates in pulmonary airways during ADSV delivery, oxygen-rich gas entering via the central stream and CO_2_-laden gas returning via the peripheral stream could support simultaneous oxygenation and ventilation without requiring significant net volume change between inspiration and expiration. Wall shear forces generated by the peripheral return flow may also contribute to mucus mobilization and secretion clearance—a secondary effect potentially relevant in ARDS, where retained secretions compound ventilation-perfusion mismatch and infection risk.

This hypothesis is physiologically plausible given the known flow mechanisms that support gas exchange during high-frequency oscillatory ventilation, where mean airway pressure is maintained, and gas transport occurs despite tidal volumes smaller than dead space [14,39]. However, direct evidence for bidirectional intrapulmonary flow during ADSV-specific delivery remains unestablished. The flow patterns described here are mechanistic extrapolations from fluid-dynamic principles and device architecture, not measured in vivo or in vitro observations. Validation of the proposed flow mechanisms will require complementary computational and experimental investigations. Computational fluid dynamics and time-resolved flow visualization could characterize transient flow reversal, secondary transport, and local wall shear, while electrical impedance tomography or hyperpolarized gas MRI could assess regional ventilation distribution [40,41]. These investigations should precede clinical interpretation of the proposed gas exchange and secretion-clearance mechanisms.

## 7. Clinical Implications and Comparison with Current Ventilation Strategies

ADSV occupies a conceptually distinct position among ventilation strategies for ARDS. In conventional volume-controlled, pressure-controlled, and high-frequency modes, gas delivery is governed primarily by clinician-selected parameters generated at the ventilator and transmitted through several feet of breathing circuit before reaching the patient. This circuit distance can introduce pneumatic damping and delay between gas delivery, patient mechanical response, sensed feedback, and subsequent device adjustment. In a heterogeneous, viscoelastic lung—where recruitment and derecruitment are strongly time-dependent—this delay is physiologically relevant. By placing the sliding venturi mechanism close to the patient (less than 4 feet away), ADSV is designed to modulate subtidal gas delivery nearer to the ETT, allowing the delivered pulse to interact more directly with the instantaneous mechanical impedance of the airways and injured lung. Although the pressure–flow response occurs close to the patient, it reflects aggregate respiratory mechanics rather than independent measurements of regional pulmonary behavior. ADSV therefore proposes a local, passive, physics-driven feedback loop in which the lung’s mechanical state continuously shapes the delivered breath. The clinical implications of this distinction are clearest when ADSV is considered alongside the strategies it most closely resembles.

Compared with low-tidal-volume ventilation—the current evidence-based foundation of ARDS management—ADSV is proposed as a potential approach to addressing a limitation that lung-protective protocols have never fully resolved: the inability to achieve lung recruitment and prevent overdistension within a single breath cycle. Low-tidal-volume ventilation limits global strain by capping delivered volume, but it does not adapt to regional differences in compliance, opening pressure, or time constant behavior, and it cannot increase distending pressure in collapsed regions without risking overdistension of already-open tissue elsewhere [3,42]. ADSV’s proposed entrainment mechanism may permit changes in subtidal gas delivery in response to respiratory system impedance. Whether this response improves recruitment while avoiding regional overdistension, relative to established lung-protective ventilation, remains to be determined experimentally.

ADSV differs conceptually from high-frequency oscillatory ventilation (HFOV) in its proposed use of mechanically responsive gas entrainment and patient-proximal expiratory venting. These features may influence expiratory pressure release and mean airway pressure; however, ADSV’s effects on dynamic hyperinflation, hemodynamics, and clinical outcomes remain unestablished. Therefore, direct comparison with the outcomes of established HFOV strategies remains premature. Additionally, gas delivery in HFOV is governed by oscillation frequency, amplitude, and mean airway pressure—all of which are preset and do not inherently adapt to changing regional mechanics. In contrast to HFOV, in which the oscillatory breath is delivered according to preset ventilator settings and therefore produces a comparatively fixed subtidal volume, ADSV delivers a variable, dynamically modulated subtidal volume. ADSV’s entrainment mechanism responds to mechanical feedback from the respiratory system on millisecond timescales, continuously altering the volume entrained with each percussive pulse without an equivalent preset tidal-volume constraint. Consequently, ADSV does not prescribe a fixed subtidal volume; instead, it emerges from the interaction between the device, airway mechanics, and evolving pulmonary impedance.

The most instructive comparison is the conceptual overlap between ADSV and time-controlled adaptive ventilation (TCAV). Both strategies recognize that alveolar stability is time-dependent and that expiratory behavior—not only inspiratory pressure and volume—determines whether recruited lung units remain open. TCAV addresses this by controlling the duration of both inspiratory pressure application and expiratory release through precise timing of the airway pressure release valve [16,17]. ADSV is proposed to generate progressive pressure and volume accumulation through successive subtidal pulses with brief intervening expiratory intervals. Although this mechanism shares conceptual features with TCAV’s time-dependent recruitment philosophy, the two approaches differ in how they control pressure, flow, and expiratory timing. Their physiological interactions and potential combined applications remain subjects for future investigation.

A secondary clinical benefit proposed for ADSV is enhanced secretion clearance. If confirmed experimentally, the bidirectional flow pattern described in Section 6 would generate oscillatory wall shear forces along the airway epithelium, potentially mobilizing mucus more effectively than the unidirectional bulk flow of conventional ventilation. This effect would be directly relevant in ARDS, where impaired mucociliary clearance, secretion retention, and secondary infection are common complications that compound the primary lung injury [42,43]. Because ADSV employs externally generated, time-cycled gas pulses, the proposed delivery mechanism does not inherently require spontaneous inspiratory effort. In principle, this could permit application during passive mechanical ventilation, including in patients receiving neuromuscular blockade. However, the ability to provide adequate ventilation and oxygenation under these conditions, together with the necessary monitoring and safety requirements, has not been established clinically for the device configuration described here [18].

The proposed benefits discussed here are based primarily on device architecture, established fluid-dynamic principles, and physiological hypotheses rather than direct ADSV-specific clinical evidence. Although related high-frequency percussive ventilation systems have been investigated clinically, the available sources do not establish human clinical validation of the specific ADSV configuration examined in this article. Beyond ARDS, the proposed mechanically responsive characteristics of ADSV may have potential applications in other conditions involving altered respiratory compliance, airway resistance, or secretion clearance, including postoperative atelectasis, pulmonary edema, and obstructive airway diseases. However, these potential applications remain theoretical, and their physiological benefits and clinical suitability require dedicated experimental investigation.

## 8. Conclusions

ADSV is a proposed mechanically responsive ventilation strategy in which jet-induced gas entrainment and downstream respiratory pressure loading may modulate subtidal volume delivery. Building on previously described ADSV principles, this analysis clarifies the fluid-dynamic basis of this response and distinguishes global respiratory mechanical adaptation from regional physiological effects that remain to be established.

The physiological rationale for ADSV is grounded in established fluid-dynamic principles and the proposed coupling between subtidal gas delivery and respiratory system mechanics. Progressive pressure accumulation and patient-proximal expiratory venting may influence lung inflation, recruitment, and gas transport. However, their ability to reduce regional overdistension, prevent derecruitment, or mitigate ventilator-induced lung injury has not yet been demonstrated specifically for ADSV. The proposed bidirectional intrapulmonary flow pattern may also contribute to gas exchange and secretion clearance, but requires direct experimental confirmation.

Further investigation is needed to establish ADSV’s physiological and clinical implications. Priority studies include bench characterization of entrainment and delivered FiO_2_ under varying respiratory system impedances; measurements of regional inflation and recruitment in heterogeneous lung models; computational and experimental assessment of intrapulmonary flow; and evaluation of expiratory pressure, device reliability, and gas exchange. These studies would provide the necessary foundation for subsequent preclinical and clinical assessment of ADSV safety and efficacy.

## Figures and Tables

**Table 1:** ADSV mechanisms and experimental validation requirements.

Proposed Mechanism	Quantities to Measure	Validation Approach
Variable gas entrainment	Entrainment ratio, delivered pulse volume, downstream pressure	Bench testing with independently varied resistance and compliance
Differential inflation in heterogeneous lungs	Compartmental volume, pressure, regional strain	Two-compartment lung model with different compliance and opening pressures
Progressive subtidal pressure accumulation	Airway pressure, net volume accumulation, end-expiratory pressure	Time-resolved pressure and flow measurements
Gas mixing and oxygen delivery	Delivered FiO_2_, primary and entrained flow rates	Controlled oxygen-mixing experiments
Pulsatile gas transport	Transient velocity profiles, reversal patterns, mixing	CFD and flow visualization
Expiratory performance	Expiratory pressure, flow decay, residual lung volume	Bench testing over varying expiratory time constants
Device reliability	Valve displacement, flow obstruction, pressure response	Repeated cycling under controlled humidity and secretion-surrogate exposure

## Data Availability

No new data was created or analyzed in this study. Data sharing is not applicable to this article.

## Supplementary Materials

The following supporting information can be downloaded at https://www.mdpi.com/article/10.3390/jcm15218714/s1, Video S1: Animation of a representative ADSV sliding venturi device.
